# Computing semantic similarity of texts based on deep graph learning with ability to use semantic role label information

**DOI:** 10.1038/s41598-022-19259-5

**Published:** 2022-08-30

**Authors:** Majid Mohebbi, Seyed Naser Razavi, Mohammad Ali Balafar

**Affiliations:** Department of Computer Engineering, Faculty of Electrical and Computer Engineering, University of Tabriz, Tabriz, 51666-16471 Iran

**Keywords:** Computational science, Computer science, Information technology

## Abstract

We propose a deep graph learning approach for computing semantic textual similarity (STS) by using semantic role labels generated by a Semantic Role Labeling (SRL) system. SRL system output has significant challenges in dealing with graph-neural networks because it doesn't have a graph structure. To address these challenges, we propose a novel SRL graph by using semantic role labels and dependency grammar. For processing the SRL graph, we proposed a Deep Graph Neural Network (DGNN) based graph-U-Net model that is placed on top of the transformers to use a variety of transformers to process representations obtained from them. We investigate the effect of using the proposed DGNN and SRL graph on the performance of some transformers in computing STS. For the evaluation of our approach, we use STS2017 and SICK datasets. Experimental evaluations show that using the SRL graph accompanied by applying the proposed DGNN increases the performance of the transformers used in the DGNN.

## Introduction

The problem of similarity learning is a significant issue in pattern recognition. The goal of similarity learning is to learn a measure to reflect the semantic distance according to a specific task^1^. Similarity learning includes looking for similarity patterns to find complicated and implicit semantic patterns. Similarity learning in the text area is studied in the STS computation field. STS measures the degree of semantic overlap between two texts^2^. The ability to determine the semantic relationship between two texts is an integral part of machines that understand and infer natural language^3^ hence STS is a directly or indirectly significant component of many applications such as information retrieval^4^, recognition of paraphrases^5^, textual entailment^6^, question answering^7^, text summarization^8^, measuring the degree of equivalence between a machine translation output and a reference translation^9^ and also text summarization evaluation, text classification, document clustering, topic tracking, essay scoring, short answer scoring, etc. STS is also closely related to paraphrase identification and textual entailment recognition. Numerous research studies have been carried out on computing semantic similarity score between two sentences. The goal of the research studies in these fields is to construct a system that is able to predict the results having maximum adequateness with those assigned by human annotators. Due to the limited amount of available annotated data, variable length of sentences, and complex structure of natural language, computing semantic similarity remains a hard problem^10^. An effective step was taken by computing word embeddings^11^. Using word embeddings has led to valuable results in various Natural Language Processing (NLP) tasks. In recent years, in deep learning models, a variety of approaches have been proposed. These models have different architectures; therefore, their powers to detect implicit patterns for recognizing similarity are different. Some models utilized linear structure based on Recurrent Neural Network (RNN) architecture including Long Short-Term Memory (LSTM) and Gated Recurrent Unit (GRU) models^6,10,12–14^; some of them use a grammar tree accompanied by input text. However, it was not clear how to effectively capture the relationships among multiple words of a sentence in such a way that yields the meaning of the sentence. Efforts to obtain embeddings for larger chunks of text had not been so successful^15^. The NLP community had not found the best supervised approach for embedding that captures the semantics of a whole sentence^15^. With the introduction of BERT^16^, the design of a new generation of powerful models has begun. These models are collected under the name of *Transformer*s such as BERT, RoBERTa^17^, etc. Transformers provide general-purpose architectures for natural language understanding and natural language generation^18^. Transformers are trained on a large corpus while handling long-range dependencies between input sequences and output sequences and they can capture the meaning of the sentence effectively. They are trained on a specific dataset to be adapted for the specific task. Transformers have high power in detecting implicit patterns, so they produce state-of-the-art results in most tasks including STS.

Along with the computational power of transformers, there are supplementary representations that can be used for processing in neural networks. These representations are generated by systems developed in the typical field of NLP including dependency grammar (DG). Various models based on DG have been presented in computing STS and semantic relations tasks including Tree-LSTM^19^, recursive autoencoders^20^ and tree-structured attentive encoder^21^. The most important feature that the DG offers is the graph structure of the text, which can be used in neural networks.

Another representation is produced by Semantic Role Labeling (SRL). SRL systems operation falls into the semantic domain. SRL process assigns semantic roles to words or phrases in a sentence and produces predicate-argument structures. In most cases predicates are verbs, and arguments are phrases that determine essential details including "who?", "did what?", "with what?", "to whom?", "where?", "when?", etc. State-of-the-art systems lack any explicit modeling of SRL system output for computing STS. One potential reason why SRL system output has not been used for computing STS is the lack of effective methods for applying and incorporating SRL information in neural networks.

Our goal is to propose a method for applying SRL information and to provide a model with access to rich SRL information so that it could make a decision about which aspects of SRL information are beneficial for STS computation. SRL system output does not have a graph structure thus, it has significant challenges in dealing with graph-neural networks. We propose a novel SRL graph by using the semantic roles generated by SRL system and graph structure obtained from DG. We intend to use the computational power of graph-neural networks to process the SRL graph hence, we propose a DGNN based g-U-Net^22^ by devising Graph-Convolutional Network (GCN) to extract information from the SRL graph and to achieve our desired performance. GCNs are multilayer neural networks that have been developed to process graph data and they are able to compute the representation of a vertex based on the *k*th neighboring nodes' representations.

We extract a sequence of representations from the GCN layers of the DGNN. These representations have a similar function to Weisfeiler–Lehman^23^ sequence of graphs, except that the representations are produced by neural networks instead of kernels. These representations capture label information of vertices and edges in the SRL graph to be used to produce a prediction. To compute these representations, we generate a score for each vertex by assigning scores to the edges of each vertex in the SRL graph. This approach attempts to find a pattern that is matched for all of sentences to assign scores to the edges to compute a score for each vertex. Each vertex's score shows a degree of participation vertex's representation in the formation of the result. Since transformers have provided state-of-the-art results, our goal is to incorporate information obtained from the semantic roles in the SRL graph into representation vectors generated by transformers, and thus, potentially improve the quality of the STS computation output. To do this, we put the layers of our proposed network on top of the transformer layers to be able to use the power of transformers. The DGNN learns with a semi-supervised strategy to utilize both the representations obtained from a transformer and labeled data. The DGNN deeply mines the relationship of the different structures to improve the performance of semi-supervised regression. Since the DGNN operate on the SRL graph and uses a transformer, we are eager that our proposed DGNN improves the quality of predictions compared with the bare transformer used in the model. Experimental evaluations show that our proposed DGNN operating on the SRL graph improves Pearson and Spearman correlation coefficients of the bare transformer used in the DGNN for STS2017^2^ dataset and Sentences Involving Compositional Knowledge (SICK)^24^ dataset.

Our contributions can be summarized as follows:

A. we introduce an approach for computing semantic similarity of texts by utilizing semantic role label information.
B. we introduce a method for constructing the SRL graph based on the SRL process output and DG.
C. we offer a deep graph neural network placed on top of a transformer’s layers to process the SRL graph.
D. we show that creating the SRL graph is beneficial for STS computation on STS2017 and SICK datasets.

## Materials and methods

In this section, we offer our proposed method for constructing the SRL graph based on the SRL process output and DG. Then we present g-U-Net as the basic architecture and we offer our proposed network for processing the SRL graph.

### SRL graph

**Figure 1:**
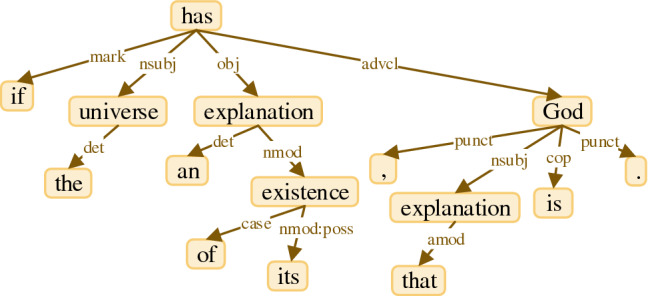
Graph structure of an example produced by Stanford dependency grammar.

One of the useful information obtained from a text is DG describing grammatical relations between words. The graph structure is a popular structure for data. This structure determines how the vertices connect to each other. Each vertex or edge can have its own representation. In the graph structure of DG, the vertices are words and the edges having grammatical labels connect the governor vertices to the dependent vertices. This representation is very suitable for use in neural networks because various operations on the representation of vertices can be easily run. By using the graph structure obtained from a DG, words can be accessed and processed beyond the linear arrangement of the words in a text. To depict the graph structure of DG of a text, we take the first text of the 2196th pair from STS2017 train set, "If the universe has an explanation of its existence, that explanation is God.". Figure 1 shows the graph structure of DG for the selected example that is provided by Stanford dependency grammar^25^ implemented in Stanford Core NLP^26^.

**Figure 2:**
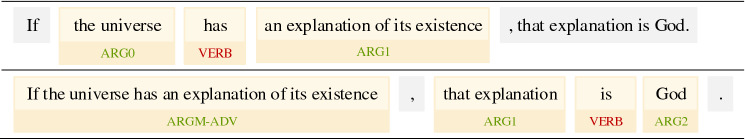
SRL system output, first and second rows illustrate arguments of first and second predicates respectively. Predicates are shown with red labels.

A piece of information obtained from a text is predicate-argument structures produced by an SRL system. By performing an SRL system, semantic roles are assigned to predicates and arguments. We use an SRL system proposed by Shi and Lin^27^ and implemented in AllenNLP^28^. Figure 2 shows the output of the SRL system for the selected example. In Fig. 2, two predicates including "*has*" and "*is*" along with their arguments are illustrated.

Our goal is to generate an SRL graph by using the semantic roles assigned to arguments and also the graph structure obtained from the DG. We propose 4 steps for generating an SRL graph.

#### Step 1: Converting each predicate-argument structure to an SRL initial graph

**Figure 3:**
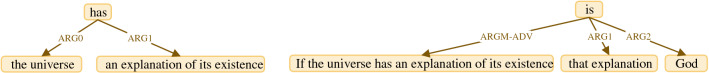
SRL initial graphs.

In this step, we set the predicate as root vertex and arguments as children that are connected to their predicate with an edge. We assign the role of each argument to the edge connecting the same argument to its predicate. Figure 3 shows two initial graphs based on the predicates and the arguments illustrated in Fig. 2. As shown in Fig. 3, each predicate is identified as a root and the arguments are identified as children connected to their own predicates.

#### Step 2: Extracting the graph structure of each argument

**Figure 4:**
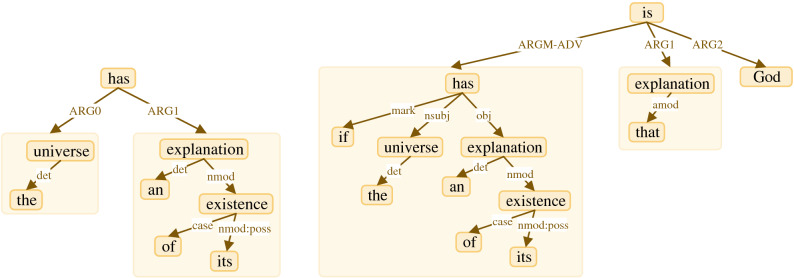
The subgraph of the arguments illustrated in Fig. 3.

In this step, we use DG to specify the graph structure of the arguments. Our goal is to select a token or tokens as a root vertex or root vertices in each argument. The root means a vertex that its father is not inside the corresponding argument. To do this, we need to extract the graph structure between the tokens in the argument; hence, we connect two tokens in the argument which are connected in the DG graph with an edge. With respect to existing tokens in the argument, it is possible to obtain more than one subgraph. Figure 4 shows the graph structures of the arguments illustrated in Fig. 3.

#### Step 3: Building a base graph

**Figure 5:**
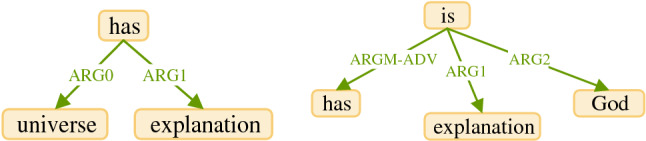
SRL base graph.

In this step, we construct a base graph by specifying the roots of the subgraphs extracted for each argument. In each argument, we hold the roots of the subgraphs and eliminate the remaining vertices. Then we connect the destination of the edge placed between a predicate and an argument to the roots obtained for the corresponding argument. As a result, for each semantic role produced by the SRL system, there will be at least one edge connecting the corresponding predicate to the root or roots obtained for its argument. Figure 5 shows the constructed base graph based on the semi graph illustrated in Fig. 4.

#### Step 4: The final step of making an SRL graph

**Figure 6:**
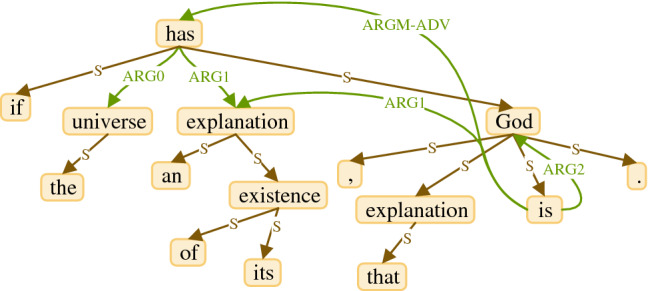
SRL + SDG graph.

At this point, we construct an SRL graph. We use the graph structure of the graph obtained from the DG. We use the DG graph in two forms; the DG graph with only one label for all of the edges and the DG graph with ordinary form. To use the former form, we replace the label of all edges with one label. Figure 6 shows all edges obtained from the DG graph have label *S* ('*S*' stands for '*Simple*'). This indicates that all of these edges belong to the DG graph and have the same type. Then, we add the edges of the base graph generated in the previous step to this graph. If an edge in the base graph connects two vertices that were connected by one *S* edge, the *S* edge is replaced with the edge of the base graph. We denominate this SRL graph as SRL + SDG. In Fig. 6, the edges of the base graph are added to the graph structure to form the final SRL graph. To use the ordinary form of DG graph, we repeat the same steps for construction SRL + SDG, except that we do not change the labels of the edges of DG graph. We denominate this SRL graph as SRL + DG. As shown in Fig. 6, adding the edges of the base graph to the graph structure obtained from DG changes the graph structure and changes information flow in the graph, consequently.

### G-U-Net

The U-Net architecture^29^ is highly regarded due to its high performance. This architecture is generally suitable for grid-like data such as images. Gao and Ji^22^ proposed graph-U-Net (g-U-Net) architecture by utilizing the U-Net architecture accompanied by GCN and introducing the graph pooling (gPool) and graph unpooling (gUnpool) layer. G-U-Net has several GCN and gPool layers on the decoder side. Several gUnpool layers corresponding with gPool layers are on the encoder side. GCN itself can have various architectures to produce a new representation for the vertices and edges. gPool layer generates a score for each vertex in the graph and selects some vertices based on selecting k-max scores, and produces a smaller graph by eliminating the remaining vertices corresponding to unselected scores. gUnpool layer operates on the opposite of the corresponding gPool layer. It defines a zero matrix with the same size as the graph which is fed into the corresponding gPool layer and restores the selected vertices to the zero matrix by using selected indexes, then gUnpool layer imports all the representations transmitted through skip connection to the produced matrix.

To be able to use the features of U-Net on the graph structure, we propose a g-U-Net based architecture.

### The proposed DGNN’s architecture

**Figure 7:**
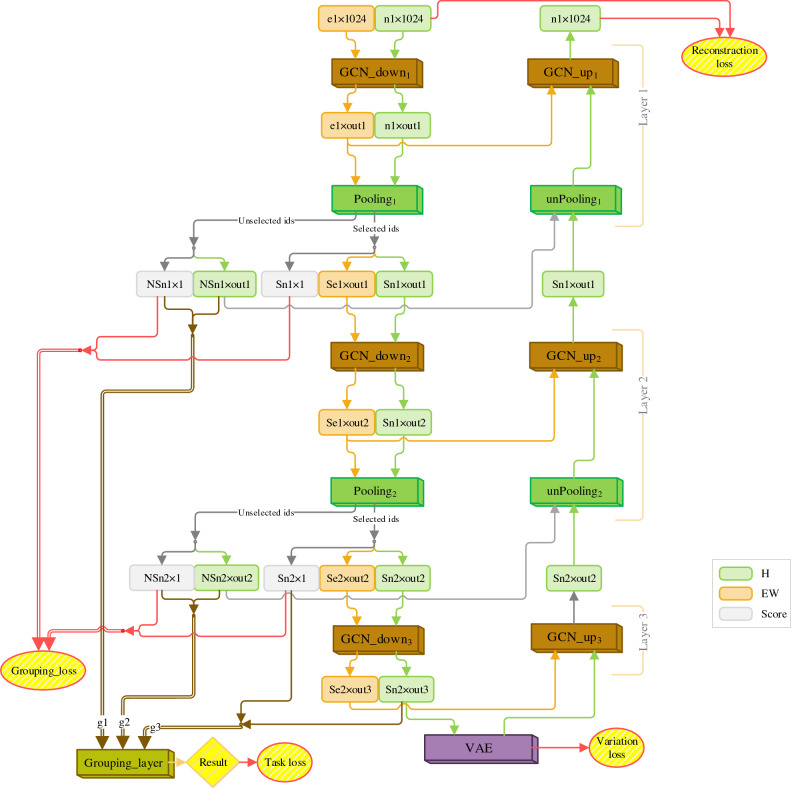
An illustration of our proposed DGNN’s architecture in 3 layers.

Our proposed DGNN is organized based on graph-U-Net’s architecture with an optional number of layers to produce a new representation for each vertex and edge in each GCN layer. By using graph-U-Net’s architecture, the DGNN can extract a sequence of representations to produce a prediction commensurate with a specific task. We have several components including GCN, Pooling, and unPooling because of the use of the graph-U-Net’s architecture. The DGNN’s architecture has two sides, an encoder and a decoder; accordingly, each layer in this architecture includes these two sides. On the encoder side, each layer contains a GCN layer and a Pooling layer, and on the decoder side, each layer contains an unPooling layer and a GCN layer. Figure 7 illustrates the DGNN’s architecture in 3 layers. Each GCN layer has a down_j_ or up_j_ label to specify the encoder or decoder side respectively. Indicator *j* indicates layer number. The last layer doesn’t have any pooling layer; it only contains the GCN layer.

In the $j$th layer, *GCN_down*_*j*_ takes $H$ and $EW$ as inputs. $H$ contains the representations produced by the transformer and $EW$ contains the representations for the edges. $H$ and $EW$ are described in detail in Supplementary Sect. 1. Then *GCN_down*_*j*_ processes the entire graph and outputs $H \mathrm{and }EW$ as new representations for all of the vertices and edges. *Pooling*_*j*_ takes the output of *GCN_down*_*j*_ and assigns a score to each edge; then *Pooling*_*j*_ produces a score for each vertex by using the scores of the edges belonging to the vertex. Based on the generated score for each vertex, *Pooling*_*j*_ divides the index of the vertices into selected and unselected indices. The loss of this division is computed by *Grouping_loss*. Unselected vertices and their associated edges are removed from the output of *GCN_down*_*j*_ to form a smaller graph. The smaller graph is sent to the *GCN_down*_*j*+*1*_. In Fig. 7*outj* is the size of the representation generated by *GCN* in the *j*th layer. *Snj* and *Sej* are numbers of selected vertices and edges respectively in the *j*th layer. *NSnj* is number of unselected vertices in the *j*th layer.

In each layer of the proposed U-Net, the representation of unselected vertices along with their generated scores are sent to *Grouping_layer*. *unPooling*_*j*_ layer has two inputs. First, the representation obtained from *GCN_up*_*j*+*1*_ belonging to the selected vertices. Second, the representation of the unselected vertices from *GCN_down*_*j*_. *unPooling*_*j*_ expands the graph obtained from *GCN_up*_*j*+*1*_ into its initial size in the *j*th layer by producing the representation based on its input representations. *GCN_up*_*j*_ takes the representation of the vertices obtained from *unPooling*_*j*_, and the edge representations from *GCN_down*_*j*_ to produce a new representation for each vertex.

We apply Variational Autoencoders (VAE)^30^ to process the representations moved from the encoder side to the decoder side in the last layer. Intuitively, the VAE learns representations as soft ellipsoidal regions in latent space instead of single points. VAE forces the representations to fill the space rather than memorizing the training data as single representations^31^. Here, we define ${loss}_{VAE}$ similar to the loss defined by Kingma and Welling^30^. As well as we define ${loss}_{reconstraction}$ in Eq. (1).

1 $${loss}_{reconstraction}=SmoothL1Loss(output,input),$$

where, $input$ is the input of *GCN_down*_*1*_ in the first layer (the representations obtained from a transformer), and $output$ is the output of *GCN_up*_*1*_ in the first layer.${loss}_{reconstraction}$ causes the DGNN learns with a semi-supervised strategy to utilize both the representations obtained from a transformer and labeled data. ${loss}_{reconstraction}$ uses *SmoothL1Loss*^32^ to bring the representation of $input$ and $output$ closer together. These losses are used to calculate the total loss, which is described in Supplementary Sect. 7.

Supplementary Sects. 1–7 describe all of the components of the DGNN’s architecture in detail.

## Results

In this section, we introduce the datasets and transformers used in the proposed DGNN and then present the results of the experimental evaluations.

### DataSet

We use two datasets to evaluate the DGNN.

- STS2017 benchmark is provided to evaluate STS systems. STS2017 consists of 8628 sentence pairs. The sentence pairs were split up into 5749, 1500, 1379 for train, dev, and test sets respectively. STS is collected from the three categories captions, news, and forums. The label of each sentence pair is a score in the interval [0.0, 5.0]. Score 0.0 indicates there is no relation between sentences in a pair, and score 5.0 indicates sentences are semantic equivalence. We evaluate the DGNN on STS2017 using Pearson and Spearman correlation coefficients.
- SICK had been offered by Marelli et al.^24^. SICK consists of 9927 sentence pairs. The sentence pairs were split up into 4500, 500, 4927 for train, dev, and test sets respectively. These sentence pairs had been collected from image and video description datasets. The label of each sentence pair is a score in the interval [1.0, 5.0]. Score 1.0 indicates that the two sentences are completely unrelated, and score 5.0 indicates that the two sentences are wholly related. Similar to STS2017 benchmark, we evaluate the DGNN on SICK using Pearson and Spearman correlation coefficients.

### Transformers

Abundant transformers have been proposed such as BERT, RoBERTa, etc. In order to reduce the workload in this article, we only use BERT and RoBERTa in our experiments and refuse to utilize the other transformers. BERT is a bidirectional transformer that computes representation by jointly conditioning on both left and right contexts in all layers. BERT was offered with 12 and 24 layers. BERT uses a self-attention mechanism^33^ in each layer. In the training phase, BERT combines masked language modeling (MLM) objective and next sentence prediction (NSP). RoBERTa proposed the same architecture as BERT. In RoBERTa, NSP objective is omitted, hyperparameters are modified which have a significant impact on the final results, and larger mini-batches are chosen for training.

We use the transformers library^18^ in Pytorch. We use BERT with *bert-base-uncased* config. The general characteristics of this config are as follows,

- 12-layer,
- 768-vector dimension,
- 110M parameters.

We use BERT with *bert-large-cased* config. The general characteristics of this config are as follows,

- 24-layer,
- 1024-vector dimension,
- 335M parameters.

We use RoBERTa with *roberta-large-mnli* config. The general characteristics of this config are as follows,

- 24-layer,
- 1024-vector dimension,
- 355M parameters
- fine-tuned on MNLI^34^.

We put our proposed layers on top of the transformer’s layers.

For the test phase, we pick the weights of the model with the highest mean of Pearson and Spearman for development set of STS2017 and SICK.

### Experiments

1. Determining the number of GCN layers in the DGNN’s architecture

Since the representations of the *k*th hop neighborhoods affect the computation of the representation of a vertex in the proposed GCN, determining the number of layers is important. Hence, we must determine the number of layers in order to achieve the highest efficiency. We examine the efficiency of the DGNN with different layers over SRL + SDG, SRL + DG, and DG graphs to determine the appropriate number of layers for each graph. We train the DGNN with several layers on top of RoBERTa (*roberta-large-mnli* config) on STS2017 dataset. Table 1 shows the results of the DGNN on the dev part of STS2017 over SRL + SDG, SRL + DG, and DG graphs.

**Table 1:** Results of the DGNN with various numbers of the levels on top of RoBERTa over **SRL + SDG**, **SRL + DG**, and **DG** graphs in terms of Pearson/Spearman on STS2017/Dev.

	SRL + SDG		SRL + DG		DG	
	Pearson	Spearman	Pearson	Spearman	Pearson	Spearman
**RoBERTa**						
+ 2-layer system	0.9263	0.9251	0.9256	0.9239	0.925	0.9236
+ 3-layer system	0.9258	0.9249	0.9272	0.9261	0.9243	0.9233
+ 4-layer system	0.9267	0.9253	0.9272	0.9259	0.9275	0.9264
+ 5-layer system	0.9232	0.9214	0.9282	0.9268	0.9235	0.9233
+ 6-layer system	0.9217	0.9225	0.9251	0.9237	0.9210	0.9201

According to Table 1, the results of the DGNN with 4 layers on the SRL + SDG graph have the highest performance. Also, the proposed approach with 5 layers and 4 layers has the highest performance over the SRL + DG and the DG graph respectively.

2. Comparing SRL + SDG, SRL + DG, and DG graphs

**Figure 8:**
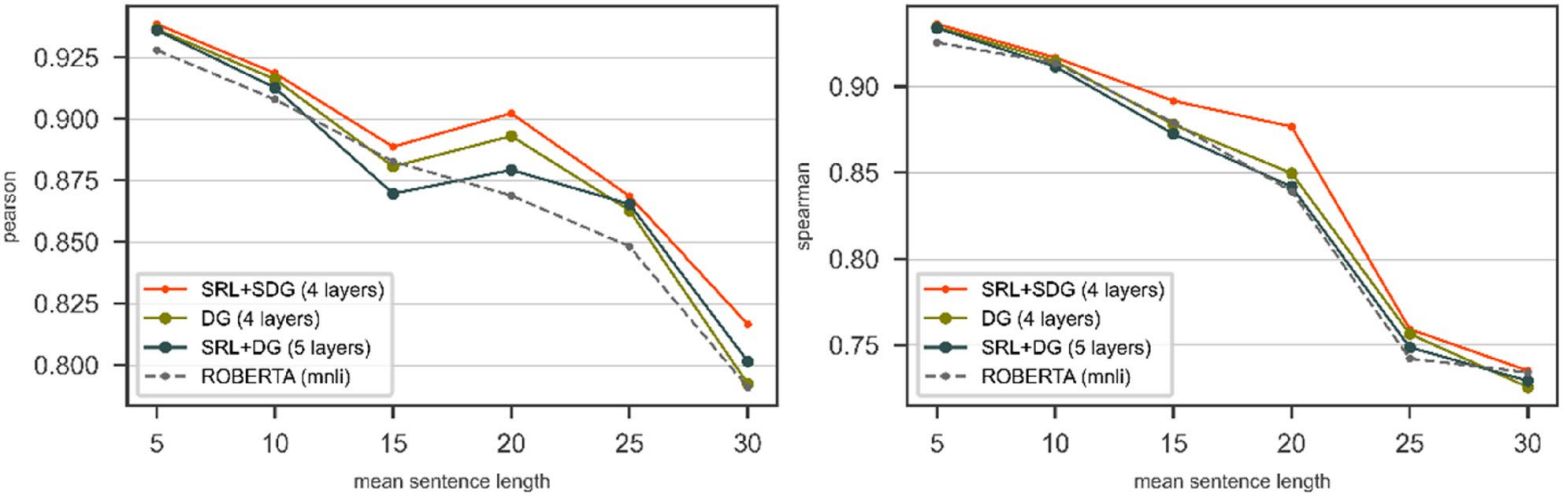
The results of RoBERTa and the DGNN on top of the RoBERTa over SRL + SDG, SRL + DG, and DG graphs on STS2017/Test in terms of Pearson (left) and Spearman (right). The pair of sentences with mean length is categorized in the window $\left[-2, 2\right]$.

By specifying the number of layers for each graph, we examine the performance of the DGNN over SRL + SDG, SRL + DG, and DG graphs to determine on which graph the performance of the DGNN is higher than the others. To investigate the performance of the DGNN on sentence length, we categorize the pair of sentences with mean length in the window $\left[-2, 2\right]$. Therefore we get several categories including 5, 10, 15, and so on. We train the DGNN with the specified number of layers (see the results of the previous subsection) over SRL + SDG, SRL + DG, and DG graphs with RoBERTa (*roberta-large-mnli* config) on STS2017 dataset. Figure 8 shows the results of bare RoBERTa (*roberta-large-mnli* config) compared to the DGNN on top of RoBERTa (*roberta-large-mnli* config) over SRL + SDG, SRL + DG, and DG graph on the test part of STS2017. According to Pearson results in Fig. 8, in category 15, the results of the DGNN over SRL + DG is lower than RoBERTa, but the DGNN over DG graph offers better results than both RoBERTa and the DGNN over SRL + DG. The DGNN over SRL + SDG has the best result compared to the SRL + DG, DG, and bare RoBERTa. As shown in Fig. 8, the Spearman results of the DGNN over DG, SRL + DG, and RoBERTa almost are the same, and the DGNN over SRL + SDG offers the best Spearman results.

In short, the SRL + SDG graph offers higher performance results than the SRL + DG and DG graphs. The reason for the better results of the SRL + SDG to DG graph is the existence of additional paths created in the graph and the existence of more efficient information flow in the SRL + SDG graph.

Due to the better results of using the SRL + SDG graph than the rest, in other experiments, we leave out the SRL + DG and DG graphs and examine the DGNN only with the SRL + SDG graph.

3. Using the SRL + SDG graph on SICK dataset

**Figure 9:**
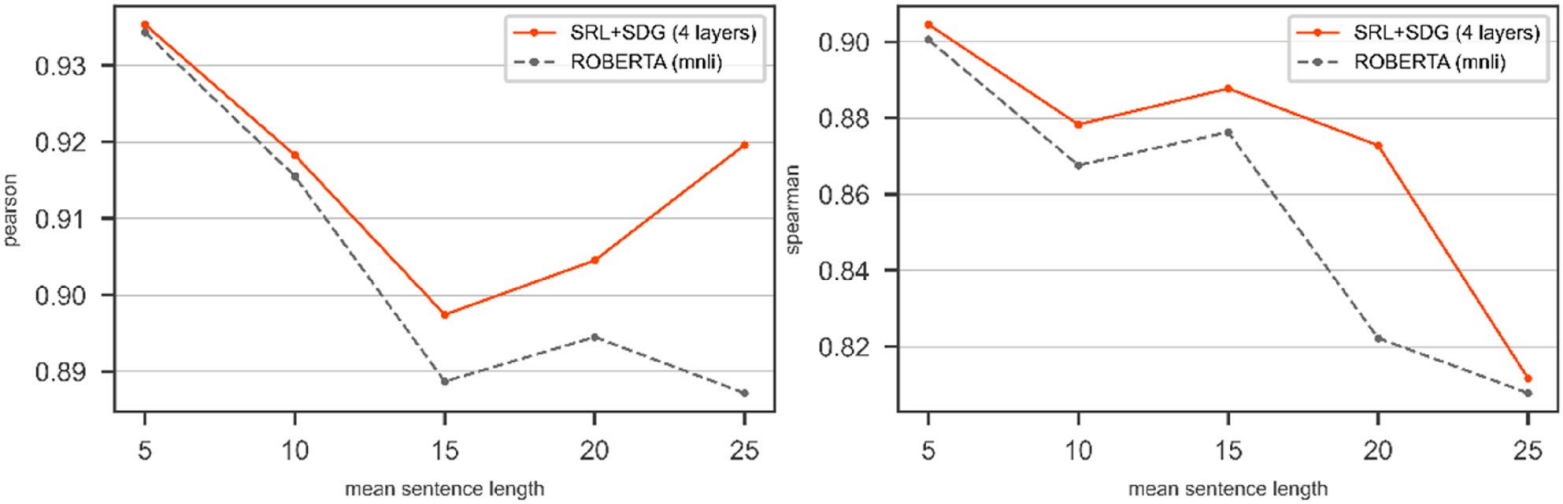
The results of RoBERTa and the DGNN on top of the RoBERTa over SRL + SDG on SICK/Test in terms of Pearson (left) and Spearman (right). The pair of sentences with mean length is categorized in the window $\left[-2, 2\right]$.

We show the result of using the SRL + SDG graph on SICK dataset. We train the DGNN over SRL + SDG with RoBERTa (*roberta-large-mnli* config) on SICK dataset. Figure 9 shows the results of bare RoBERTa (*roberta-large-mnli* config) compared to the DGNN on top of RoBERTa (*roberta-large-mnli* config) over SRL + SDG graph on the test part of SICK.

Figure 9 shows the Pearson and Spearman curves of the DGNN over the SRL + SDG graph are placed upper than RoBERTa's curves in each point of mean sentence length. These results indicate that the use of the DGNN over SRL + SDG graph improves the quality of RoBERTa's results.

4. The SRL + SDG graph on top of the other transformer

**Figure 10:**
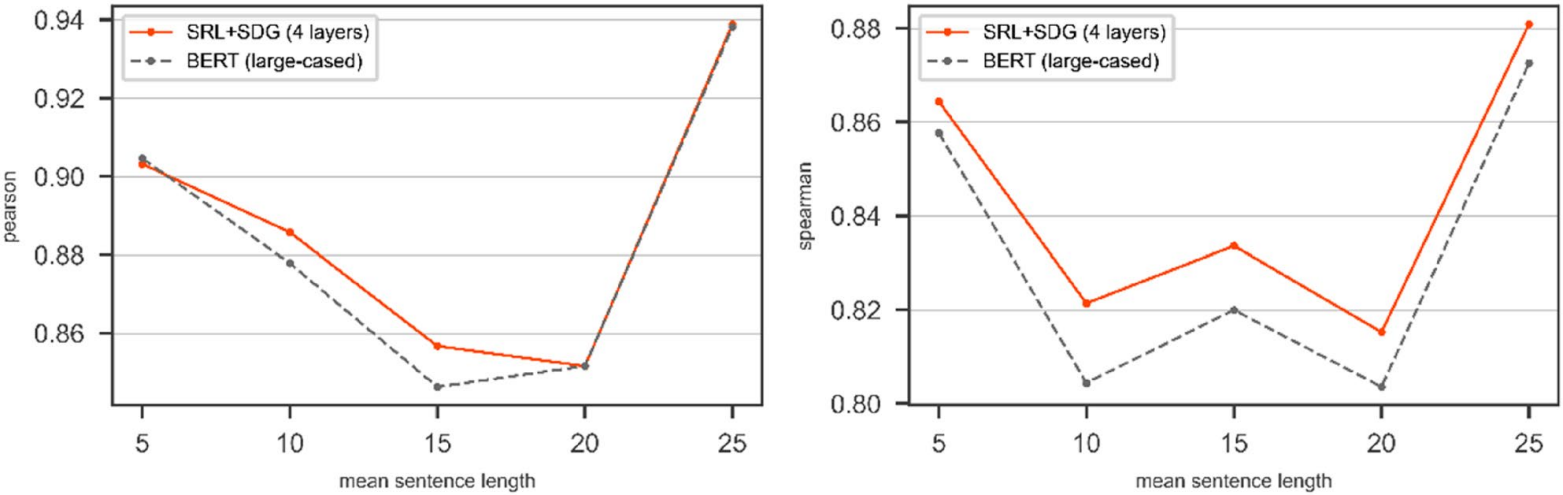
The results of BERT with *bert-large-cased* config and the DGNN on top of the BERT on SICK/Test in terms of Pearson (left) and Spearman (right). The pair of sentences with mean length is categorized in the window $\left[-2, 2\right]$.

**Figure 11:**
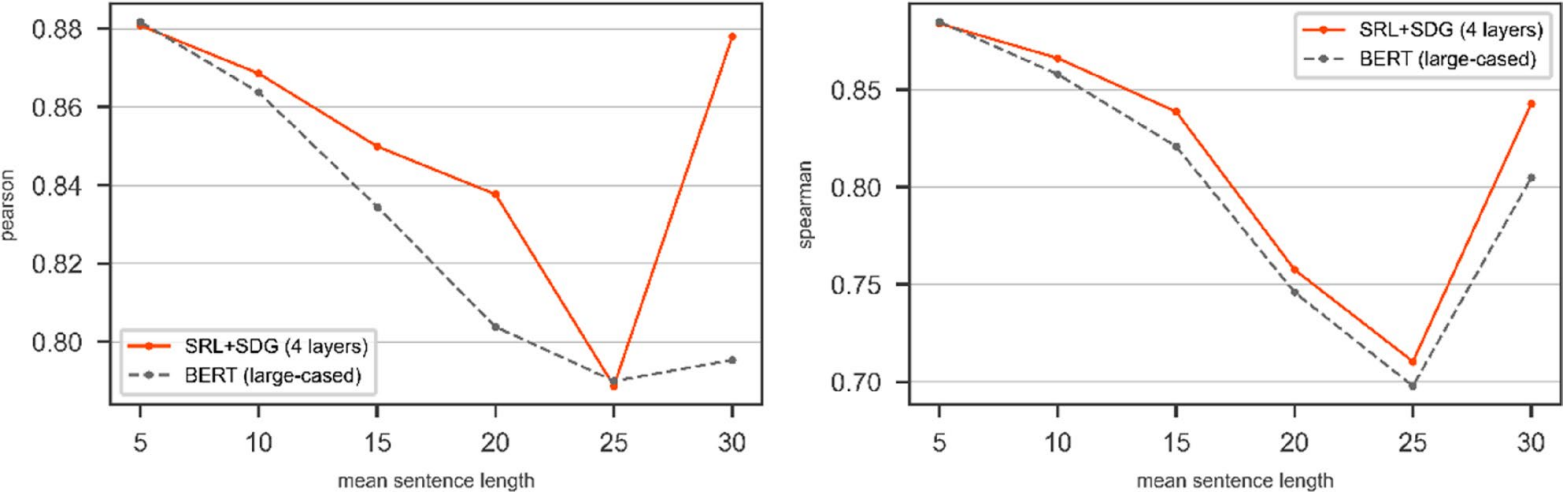
The results of BERT with *bert-large-cased* config and the DGNN on top of the BERT on STS2017/Test in terms of Pearson (left) and Spearman (right). The pair of sentences with mean length is categorized in the window $\left[-2, 2\right]$.

We use the SRL + SDG graph over STS2017 and SICK datasets on the other transformer. We want to compare the results of a bare transformer compared to the DGNN on top of that transformer. We use BERT with *bert-large-cased* config as a transformer in our experimental evaluations. The results of bare BERT (*bert-large-cased*) compared to the DGNN on top of BERT (*bert-large-cased*) over SRL + SDG graph on the test part of SICK and STS2017 are shown in Figs. 10 and 11 respectively.

Figures 10 and 11 show the use of the SRL + SDG graph with the DGNN improves the Pearson and Spearman correlation coefficients of the bare transformer in the most point of mean sentence length for both SICK and STS2017 dataset.

**Figure 12:**
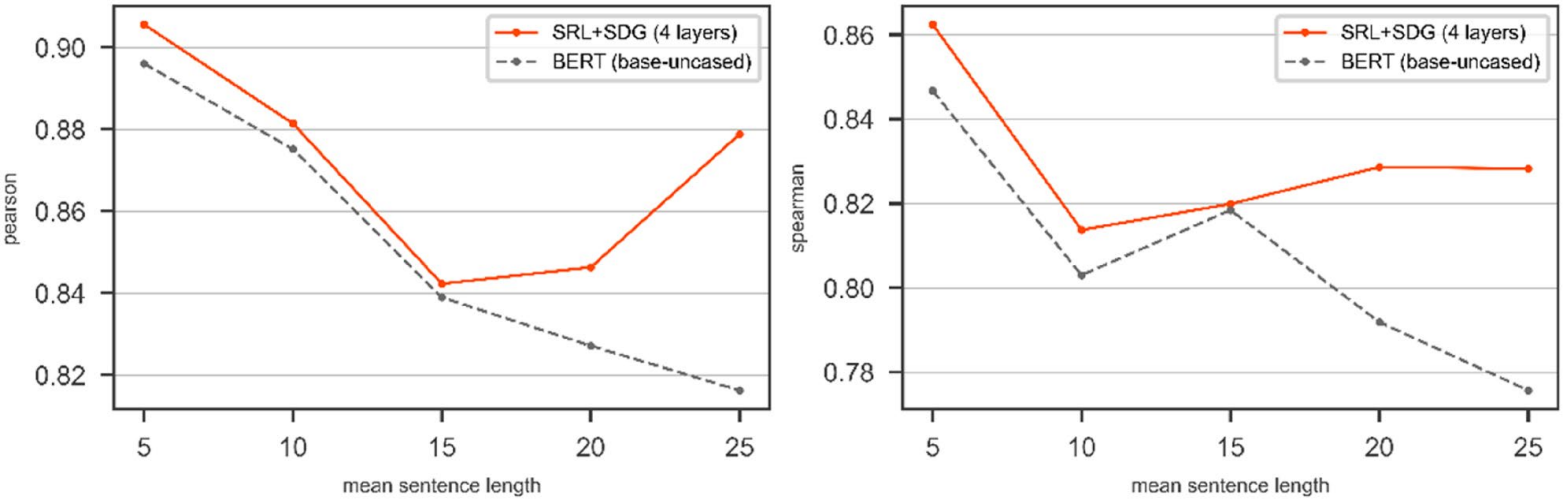
The results of BERT with *bert-base-uncased* config and the DGNN on top of the BERT on SICK/Test in terms of Pearson (left) and Spearman (right). The pair of sentences with mean length is categorized in the window $\left[-2, 2\right]$.

**Figure 13:**
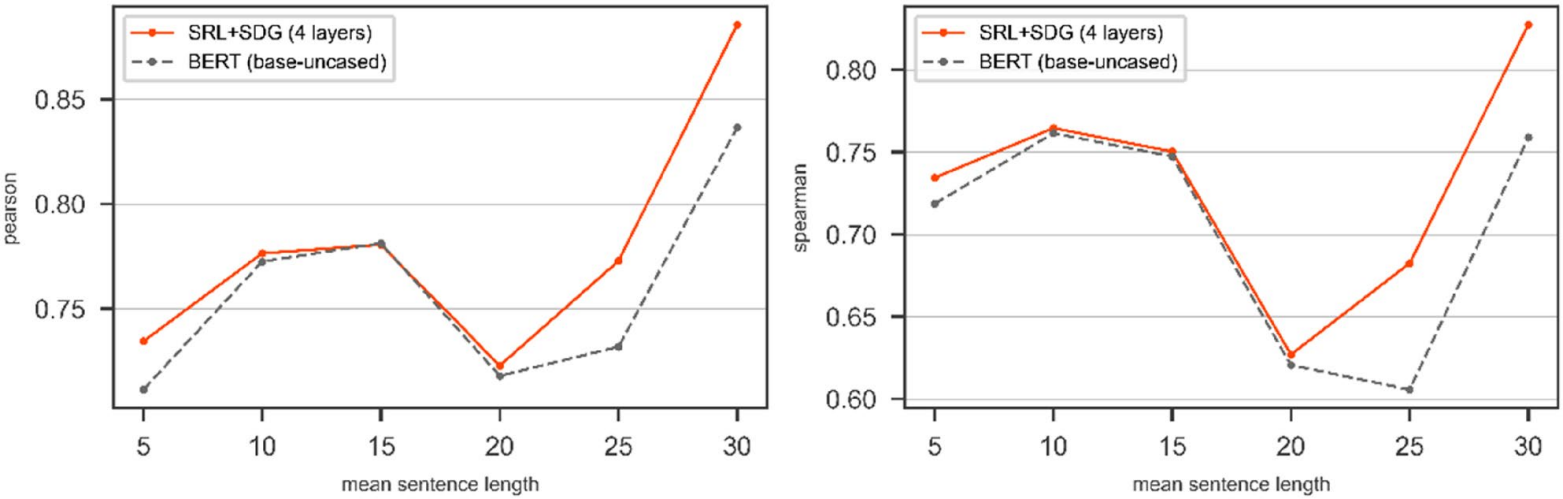
The results of BERT with *bert-base-uncased* config and the DGNN on top of the BERT on STS2017/Test in terms of Pearson (left) and Spearman (right). The pair of sentences with mean length is categorized in the window $\left[-2, 2\right]$.

In a further experimental evaluation, we use BERT with *bert-base-uncased* config as a transformer. Figures 12 and 13 show the results of bare BERT (*bert-base-uncased*) compared to the DGNN on top of BERT (*bert-base-uncased*) over SRL + SDG graph on the test part of SICK and STS2017 respectively. Figures 12 and 13 show the results of using the SRL + SDG graph with the DGNN in terms of Pearson and Spearman correlation coefficients compare to BERT with *bert-base-uncased* config.

As shown in Figs. 12 and 13 in the most point of mean sentence length, the DGNN by using the SRL + SDG graph improves the bare transformer's results for both SICK and STS2017 datasets.

## Concluding remarks and future work

In this article, we proposed SRL graph to compute STS. We introduced two types of SRL graph; SRL + DG and SRL + SDG. The SRL + DG uses the DG graph as the base graph. The SRL + SDG uses the DG graph in such a way that the type of all edges of the DG is considered as one type. We proposed a DGNN for computing the SRL graph that is placed on top of a transformer. We studied the effect of using the SRL graph on the performance of transformers on STS2017 and SICK datasets. Experimental evaluations showed that running the DGNN over the SRL + SDG graph increases the performance of those transformers compared to the SRL + DG and DG graphs. We observed that the DGNN over the SRL + SDG improves the quality of results compared with the bare transformers used in the model. We examined the degree of improvement in the quality of results for different categories of sentence lengths. Our observations showed that for one transformer and one dataset, the improvement is obtained for all categories of sentence lengths. However, by changing the transformer or the dataset, in a few categories of the sentence lengths, the obtained results are similar to the results of the transformer but in the rest of the categories, better results are produced than the transformer's results. Using the DGNN over the SRL + SDG allows us to improve the performance of the transformers to construct a system that is able to predict high-quality results.

In this research, we used DG graph to construct the SRL graph. It is suggested that future researches in this area can be an attempt to include other structure grammars for constructing the SRL graph, for example, constituency parse tree, link grammar, etc. Future research studies may focus on combining these grammars to construct a base graph and the effect of utilizing these base graphs can be studied on constructing the SRL graph to compute STS and other NLP tasks.

## Supplementary Information

Supplementary Information.

## Data Availability

The datasets for this study are available through: STS2017: http://ixa2.si.ehu.eus/stswiki/index.php/STSbenchmark. SICK: https://marcobaroni.org/composes/sick.html.
