# Supplementary material for: Computing semantic similarity of texts based on deep graph learning with ability to use semantic role label information
Source: Sci Rep. 2022 Aug 30;12:14777. doi: 10.1038/s41598-022-19259-5 (PMC9428166; doi:10.1038/s41598-022-19259-5)
Supplement: Supplementary file 1 — Supplementary Information. [file 41598_2022_19259_MOESM1_ESM.docx]

**Supplementary Information for**

**Computing semantic similarity of texts based on deep graph learning with ability to use semantic role label information**

Majid Mohebbi^1a^, Seyed Naser Razavi^1b*^, Mohammad-Ali Balafar^1c^

^1^ Department of Computer Engineering, Faculty of Electrical and Computer Engineering, University of Tabriz, 51666-16471, Tabriz, Iran

^a^Ph.D. candidate, majeedmohebbi@gmail.com

^b^Assistant Professor, n.razavi@tabrizu.ac.ir

^c^Professor, Balafarila@yahoo.com

^*^corresponding author

**Supplementary Text**

**1. Requirements**

**2. The proposed GCN**

**3. The proposed pooling layer**

**4. The proposed unpooling layer**

**5. The proposed Grouping_loss**

**6. The proposed Grouping_Layer**

**7. Computation of the total loss**

**8. Settings and hyper parameters**

**9. References**

Supplementary Sections 1 to 7 describe in detail the components of the DGNN’s architecture. Supplementary Section 8 describes settings and hyperparameters for training and test phases.

**1. Requirements**

We propose a DGNN based g-U-Net’s architecture to be able to use the features of U-Net on the graph structure. We feed input text into Stanford core NLP ^1^ to obtain tokens and the dependency graph. Also, we feed the input text into the transformer and use its output vectors as representations for the tokens. Since most transformers use Byte-Pair Encoding (BPE) technique ^2^ to make their own dictionaries, the number of output representations usually is greater than the number of tokens, because one token may be broken into several sub-tokens. We sum the representations of sub-tokens to form one representation for the token corresponding with the sub-tokens. Each token is a vertex in the SRL graph.

To specify the notation, let $G=\left\{ \left. X, E \right\} \right.$ be an SRL graph, where $X$ is the set of vertices, and $E$ is the set of directed edges; $E\in\mathbb{R}^{2\times e}$, $e$ is a number of edges in $E$, and 2 indicate two vertices of one edge. Applying this definition for $E$ allows us to use the message passing architecture created by Duvenaud et al. ^3^ in our implementation. Let $H\in\mathbb{R}^{n\times c}$ be a weight matrix for vertex, where $n$ is a number of vertices in $X, and c$ is the dimensionality of the representation. $H$ contains the representations produced by the transformer as described above. By using $H$, we can utilize the power of transformers and also tune the transformer's weights by backpropagation of error in the training phase. Let $EWS\in\mathbb{R}^{r\times c}$ be weight matrix for the edges set, where $r$ is the size of the edge-label set. $EWS$ is stored using an embedding mechanism. Since $G$ has $e$ edges, $EW\in\mathbb{R}^{e\times c}$ is weight matrix for $G$ that is selected from $EWS$ according to the labels of the edges. In the following, we describe the components of the proposed DGNN’s architecture including our proposed GCN, Pooling, and unPooling layers.

**2. The proposed GCN**

In each layer of the DGNN’s architecture, we need to produce a new representation for each vertex and edge of the input graph. We proposed a GCN for working on the SRL graph. The inputs of our proposed GCN are $H$ and $EW$. Supplementary Fig. S1 illustrates the proposed GCN architecture.

Fig. S1. An illustration of the proposed GCN architecture.

According to Supplementary Fig. S1, We compute new representations for $H$ $\mathrm{and}EW$ by using Supplementary Equation (S1) and Supplementary Equation (S2).

$H^{(j)}=\rho\left( {H^{(j-1)}\times W}_{1}^{(j)}+b_{1}^{(j)} \right),$ (S1)

${EW}^{(j)}=\rho\left( {EW}^{(j-1)}\times W_{2}^{(j)}+b_{2}^{(j)} \right),$ (S2)

Where, $W_{i}\in\mathbb{R}^{{in}_{j}\times{out}_{j}}, i\in\{1,2\}$ and $b_{i}\in\mathbb{R}^{{out}_{j}}, i\in\{1,2\}$ are trainable weight matrices. ${in}_{j}\mathrm{and}{out}_{j}$ are input and output dimension sizes in the *j*th layer, respectively. Here, activation function $\rho(\cdot)$ is tanh. $\times$ denotes matrix multiplication operator. ${EW}^{(j)}\in\mathbb{R}^{e\times{out}_{j}}$ is the representation of the edges in the $j$th layer for the SRL graph. $H^{(0)}$ and ${EW}^{(0)}$ contain the representations as described in Supplementary Section 1. In the training process, dropout is applied to $H^{(j)}$ and ${EW}^{(j)}$. Supplementary Equation (S1) and Supplementary Equation (S2) indicate two Fully Connected (FC) neural networks as shown in Supplementary Fig. S1. After applying Supplementary Equation (S1) and Supplementary Equation (S2), the dimensionality of new vectors is ${out}_{j}$. We use a symmetric normalized matrix ^4^ as defined in Supplementary Equation (S3).

$U=D^{-\frac{1}{2}}AD^{\frac{1}{2}}$, (S3)

Where, $A\in\mathbb{R}^{2\times e}$ is the adjacency matrix of the SRL graph. $D$ includes in-degree of each vertex in $A$. We have $U\in\mathbb{R}^{e}$.

Now we describe GCN core shown in Supplementary Fig. S1. We use graph structure to produce new representations for each vertex. We separate the set of edges belonging to each vertex in the SRL graph into two categories: input edges and output edges. Supplementary Fig. S2 shows the input and output edges for vertex *B* so that the destination of the input edges and the source of the output edge are vertex *B*.

Fig. S2. (1) An example graph. (2) A subgraph with input edges for vertex B. (3) A subgraph with output edges for vertex B.

Let $T^{(j)}$ be representations of the target vertices of all of the edges in the $j$th layer. These representations are selected from $H^{(j)}$ according to the target vertex of each edge. We have $T^{(j)}\in\mathbb{R}^{e\times{out}_{j}}$. By using Supplementary Equation (S4) and Supplementary Equation (S5), we produce *Ein* and *Eout* that contain new representations for all of the edges and they are used as representations for the input and the output edges respectively. *Ein* and *Eout* are produced based on the representations of edges and the target vertices of the same edges. This causes the edges to have different representations from each other while having the same labels and different target vertices.

${Ein}^{(j)}=\rho_{1}\left( T^{(j)}\times W_{3}^{(j)}+b_{3}^{(j)} \right)\times{EW}^{(j)}+\rho_{2}\left( T^{(j)}\times W_{4}^{(j)}+b_{4}^{(j)} \right),$ (S4)

${Eout}^{(j)}=\rho_{1}\left( T^{(j)}\times W_{5}^{(j)}+b_{5}^{(j)} \right)\times{EW}^{(j)}+\rho_{2}\left( T^{(j)}\times W_{6}^{(j)}+b_{6}^{(j)} \right),$ (S5)

Where, $W_{i}\in\mathbb{R}^{{out}_{j}\times{out}_{j}}, i\in\{3,4,5,6\}$ and $b_{i}\in\mathbb{R}^{{out}_{j}}, i\in\{3,4,5,6\}$ are trainable weight matrices. Here, activation function $\rho_{1}(\cdot)$ and $\rho_{2}(\cdot)$ are sigmoid and tanh respectively.

Now, we need to produce two representations for each vertex by using *Ein* and *Eout*. First, we produce a new representation for each vertex by using input edges. Let $e_{nm}\in E$ be an edge from vertex *n* to *m* and let $Ni(m)$ be a set of immediate neighboring vertices placed on input edges. We compute ${{Vin}_{m}}^{(j)}$ for vertex *m* by using input edges according to Supplementary Equation (S6)-(S8).

${{ri1}_{nm}}^{(j)}=concatenate\left( h_{m}^{(j)},{Ein}_{nm}^{(j)} \right), n\in Ni\left( m \right) and h_{m}^{(j)}\in H^{(j)}$ (S6)

${{ri2}_{nm}}^{(j)}=u_{nm}\circ\rho\left( {{ri1}_{nm}}^{\left( j \right)}\times W_{7}^{\left( j \right)}+b_{7}^{\left( j \right)} \right), u_{nm}\in U$ (S7)

${{Vin}_{m}}^{(j)}=\sum_{n\in Ni(m)} ({{ri2}_{nm}}^{(j)}),$ (S8)

Where, $W_{7}\in\mathbb{R}^{2*{out}_{j}\times{out}_{j}}$ and $b_{7}\in\mathbb{R}^{{out}_{j}}$ are trainable weight matrices and $concatenate$ operator concatenates two representations; activation function $\rho(\cdot)$ is tanh; $(\circ)$ denotes element-wise product operation. In the training process, dropout is applied to ${{ri2}_{nm}}^{(j)}$.

Second, we produce a new representation for each vertex by using output edges. Let $e_{mz}\in E$ be an edge from vertex *m* to *z* and let $No(m)$ be a set of immediate neighboring vertices placed on output edges. We compute ${{Vout}_{m}}^{(j)}$ for vertex *m* by using output edges according to Supplementary Equation (S9)-(S11).

${{ro1}_{mz}}^{(j)}=concatenate\left( h_{m}^{(j)},{Eout}_{mz}^{(j)} \right), z\in No\left( m \right) and h_{m}^{(j)}\in H^{(j)}$ (S9)

${{ro2}_{mz}}^{(j)}=u_{mz}\circ\rho\left( {{ro1}_{mz}}^{\left( j \right)}\times W_{8}^{\left( j \right)}+b_{8}^{\left( j \right)} \right), u_{mz}\in U$ (S10)

${{Vout}_{m}}^{(j)}=\sum_{z\in No(m)} ({{ro2}_{mz}}^{(j)}),$ (S11)

Where, $W_{8}\in\mathbb{R}^{2*{out}_{j}\times{out}_{j}}$ and $b_{8}\in\mathbb{R}^{{out}_{j}}$ are trainable weight matrices and activation function $\rho(\cdot)$ is tanh. In the training process, dropout is applied to ${{ro2}_{mz}}^{(j)}$.

We compute the final representation for vertex *m* in the $j$th layer by the combination of two representations ${{Vin}_{m}}^{(j)}$ and ${{Vout}_{m}}^{(j)}$ by using Supplementary Equation (S12) and Supplementary Equation (S13).

${V_{m}}^{(j)}=concatenate\left( {{Vin}_{m}}^{(j)}, {{Vout}_{m}}^{(j)} \right),$ (S12)

${H2}_{m}^{(j)}=\left( {H_{m}}^{(j)}\times W_{10}^{(j)}+b_{10}^{(j)} \right)+\rho\left( {V_{m}}^{(j)}\times W_{9}^{(j)}+b_{9}^{(j)} \right)+c_{1}^{(j)},$ (S13)

Where, $W_{9}\in\mathbb{R}^{2*{out}_{j}\times{out}_{j}}$, $b_{9}\in\mathbb{R}^{{out}_{j}}, W_{10}\in\mathbb{R}^{{out}_{j}\times{out}_{j}}, b_{10}\in\mathbb{R}^{{out}_{j}}, and c_{1}\in\mathbb{R}^{1}$ are trainable weight matrices. Activation function $\rho(\cdot)$ is tanh. ${H2}^{(j)}$ includes a new representation for each vertex in the $j$th layer, which is obtained by computing itself representation along with the representations obtained by using the *input* and *output* edges. In subsequent layers, the effect of the farther vertices is reflected in the representation of the current vertex. Hence, these representations capture information of vertices and edges in the SRL graph. The outputs of our proposed GCN are ${H2}^{(j)}$ and ${EW}^{(j)}$. In Fig. 7, GCN down and GCN up are the proposed GCN with different ${in}_{j}\mathrm{and}{out}_{j}$ features.

**3. The proposed pooling layer**

We need to generate a score for each vertex according to the representations generated by the proposed GCN (see Supplementary Section 2). In g-U-Nets architecture, gPool layer generates a score for each vertex by using a trainable projection vector to measure the scalar projection value of each vertex. Then it selects top-k score of the vertices. Instead of using a trainable projection vector, we propose a novel approach to generate a score for each vertex based on assigning a score to each edge corresponding to the vertex. This allows us to use the information obtained from input and output edges to generate a score. Here, we compute a score for all edges by using Supplementary Equation (S14) and Supplementary Equation (S15).

${EW2}^{(j)}=\rho\left( {EW}^{(j)} \right),$ (S14)

${EW3}^{(j)}=\rho\left( {EW2}^{(j)}\times W_{11}^{(j)}+b_{11}^{(j)} \right),$ (S15)

Where, $W_{11}\in\mathbb{R}^{{out}_{j}\times1}$ and $b_{11}\in\mathbb{R}^{1}$ are trainable weight matrices. Here, activation function $\rho(\cdot)$ is tanh. ${EW}^{(j)}$ is computed by the proposed GCN as described in Supplementary Section 2. In the training process, dropout is applied to ${EW2}^{(j)}$. Now to produce a score for each vertex in the $j$th layer, we use two representations $p_{m}^{(j)}$ and $q_{m}^{(j)}$. $p_{m}^{(j)}$ and $q_{m}^{(j)}$ respectively are generated for vertex *m* by using the score of input and output edges. We use Supplementary Equation (S16) to generate the representation of $p_{m}^{(j)}$.

$p_{m}^{(j)}=\sum_{n\in Ni(m)} ({{EW3}_{nm}}^{(j)}), nm\in E$, (S16)

We use Supplementary Equation (S17) to generate the representation of $q_{m}^{(j)}$.

$q_{m}^{(j)}=\sum_{z\in No(m)} ({{EW3}_{mz}}^{(j)}), mz\in E$, (S17)

We concatenate $p^{(j)}$ and $q^{(j)}$ by using Supplementary Equation (S18) and we define a score for each vertex by using Supplementary Equation (S19).

${pq}^{(j)}=concatenate\left( p^{(j)}, q^{(j)} \right),$ (S18)

$H_{score}^{(j)}={{pq}^{(j)}\times W}_{12}^{(j)}+b_{12}^{(j)},$ (S19)

Where, $W_{12}\in\mathbb{R}^{2\times1} and b_{12}\in\mathbb{R}^{1}$ are trainable weight matrices. The output of our proposed layer is $H_{score}^{(j)}$ that contains a score for each vertex in the $j$th layer.

Similar to gPool layer, we have to select a number of vertices to produce a smaller graph by removing the remaining vertices. gPool layer selects top-k score of the vertices with selection rate (𝜑) 0.5. In our proposed pooling, we use top-k selection procedure only in the first epoch of the training process, so in the first epoch we run top-k selection procedure with selection rate $\varphi_{j}$ for all layers except the last layer (The last layer of U-Net architecture doesn’t have any pooling layer); hence we must specify $number of layers-1$ 𝜑s.

After the first epoch, we use K-means clustering algorithm instead of top-k selection procedure for each layer. By using K-means clustering algorithm, we gather all vertex in 2 clusters including unselected vertices and selected vertices. To do this, we set the first and last vertex as centroids of 2 clusters, and perform iterative computations to optimize the positions of the centroids. K-means allocates every vertex vector to the nearest cluster by utilizing Manhattan distance. We set the following conditions to stop the repetition. When one of them is achieved, the optimization process will be halted.

- Manhattan distance between the current center and the previous center becomes less than 0.0001
- The number of repetitions becomes greater than 20.

The vertices in a cluster whose center score is higher than the other are identified as selected vertices. If the following conditions occur, we use top-k output instead of K-means clustering output.

- If the number of selected indices is less than the number of remaining layers.
- If the number of unselected indices is zero.

Performing top-k selection procedure in the first epoch and using K-means clustering in subsequent epochs cause we use the feature of both of them together. Using K-means clustering instead of imposing a selection rate brings more flexibility in producing results. The index of selected and unselected vertices in the $j$th layer are the output of the top-k selection procedure or K-means clustering algorithm. Unselected vertices are removed from the graph and the selected vertices are passed to the next layer.

**4. The proposed unpooling layer**

By moving to the bottom layer on the encoder side, the number of vertices of the graph in each layer is decreased, and the graph becomes smaller. Therefore, by moving to the upper layer on the decoder side in each layer, the reduced graph must be returned to its original size to finally produce the initial graph. gUnpool layer defines zero matrix as the same size as the input graph in the corresponding gPool layer and by using selected indexes restore the selected vertices in zero matrix, also use skip connection. Similar to gUnpool, we define a zero matrix with the same dimensions as gUnpool layer defines. Our proposed Pooling layer generates the index of selected and unselected vertices. By utilizing selected indexes, we restore the representation of the vertices obtained from the lower layer into the zero matrix. So far, this work is similar to gUnpool, but instead of using skip connection, we utilizing unselected indexes to restore the representation of the unselected vertices obtained from the encoder-side GCN layer into the matrix. In this case, instead of utilizing all of the representation by using skip connection, we only utilize the representation of the unselected vertices. By utilizing the index of selected and unselected vertices, the representation of each index is restored to its location.

**5. The proposed Grouping_loss**

According to Supplementary Section 3, suppose the top-k selection procedure or K-means clustering algorithm is executed and the indices of selected and unselected vertices are determined. In the $j$th layer, *Pooling_j_* sends the *score* of selected and unselected vertices to the *Grouping_loss*. *Grouping_loss* calculates the loss associated with the process of selecting and not selecting vertices. Let $y^{j}$ and $z^{j}$ include the scores of the unselected vertices and the selected vertices respectively. Since we do not have any *Pooling* layer in the last layer, nothing is sent to *Grouping_loss* from the last layer. We want the scores of the vertices to procure certain conditions; to do this, we define a specific loss function. We divide the interval [0, 1] into $J$ evenly spaced subintervals (we have a system with $J$ layers). The $j$th subinterval was denominated by $p_{j}$ corresponded to the $j$th layer. $p_{j}$ indicates subinterval $\left[ a_{j},b_{j} \right]$. For example, for a 3-layer system we have:

- $p_{1}=[0.0, 0.33]$
- $p_{2}=[0.33, 0.66]$
- $p_{3}=[0.66, 1.0]$

We limit each score in $y^{j}$ to be placed in $p_{j}$ and each score in $z^{j}$ to be greater than $b_{j}$ and less than $1.0$. Supplementary Fig. S3 illustrates score limitations for a 3-layer system. In this system, $y^{1}$, $z^{1}$, $y^{2}, and z^{2}$ are entered into *Grouping_loss*. The first line in Supplementary Fig. S3 indicates acceptable intervals for $y^{1}$ and $z^{1}$ with the green area and nonacceptable intervals with the red area. $\varepsilon$ is a small value to make a distance between borders. Similar to the first line, the second line indicates the acceptable and nonacceptable intervals for $y^{2}$ and $z^{2}$.

|  |  | $p_{1}$ | | | | | $p_{2}$ | | | | | $p_{3}$ | | | | |  |
| --- | --- | --- | --- | --- | --- | --- | --- | --- | --- | --- | --- | --- | --- | --- | --- | --- | --- |
|  |  |  | | | | |  | | | | |  | | | | |  |
|  |  | 0 | |  | 0.33 | | | |  | 0.66 | | | |  | 1.0 | |  |
| *Layer*_1_ |  | $\varepsilon$ | $y^{1}$ | | | $\varepsilon$ | $\varepsilon$ | $z^{1}$ | | | | | | | | $\varepsilon$ |  |
|  |  |  | | | | |  | | | | |  | | | | |  |
| *Layer*_2_ |  | | | | | | $\varepsilon$ | $y^{2}$ | | | $\varepsilon\varepsilon$ | $\varepsilon$ | $z^{2}$ | | | $\varepsilon$ |  |

Fig. S3. An illustration of the acceptable intervals of the scores generated by pooling layer. Acceptable and nonacceptable intervals are shown with the green and red areas respectively.

To generate the scores in the acceptable ranges, we define ${loss}_{Grouping Layer}$ in Supplementary Equation (S20),

${loss}_{Grouping Layer}=\sum_{j\in J-1} \left( \sum_{m\in{NSn}_{j}} \left( LF\left( max\left( \left( a_{j}+\varepsilon\right)-y_{m}^{j}, 0 \right) \right)+LF\left( max\left( y_{m}^{j}-\left( b_{j} -\varepsilon\right), 0 \right) \right) \right)+ \sum_{n\in{Sn}_{j}} \left( LF\left( max\left( \left( b_{j}+\varepsilon\right)-z_{n}^{j}, 0 \right) \right)+LF\left( max\left( z_{n}^{j}-\left( 1.0-\varepsilon\right), 0 \right) \right) \right) \right),$ (S20)

Where,$LF$ is a loss function such as *L1*, *L2*, or *SmoothL1Loss* ^5^. Let $m\in{NSn}_{j}$ indicates a member from unselected vertices in the *j*th layer, we have:

- $max\left( \left( a_{j}+\varepsilon\right)-y_{m}^{j}, 0 \right)$ results $y_{m}^{j}\geq\left( a_{j}+\varepsilon\right)$, in other words $y_{m}^{j}<\left( a_{j}+\varepsilon\right)$ is penalized.
- $max\left( y_{m}^{j}-\left( b_{j}-\varepsilon\right), 0 \right)$ results $y_{m}^{j}\leq\left( b_{j}-\varepsilon\right)$, in other words $y_{m}^{j}>\left( b_{j}-\varepsilon\right)$ is penalized.

Accordingly, $\left( a_{j}+\varepsilon\right)\leq y_{m}^{j}\leq\left( b_{j}-\varepsilon\right)$ is an acceptable interval.

Let $n\in{Sn}_{j}$ indicates a member from selected vertices in the *j*th layer, we have:

- $max\left( \left( b_{j}+\varepsilon\right)-z_{n}^{j}, 0 \right)$ results $z_{n}^{j}\geq\left( b_{j}+\varepsilon\right)$, in other words $z_{n}^{j}<\left( b_{j}+\varepsilon\right)$ is penalized.
- $max\left( z_{n}^{j}-\left( 1.0-\varepsilon\right), 0 \right)$ results $z_{n}^{j}\leq\left( 1.0-\varepsilon\right)$, in other words $z_{n}^{j}>\left( 1.0-\varepsilon\right)$ is penalized.

Accordingly, $\left( b_{j}+\varepsilon\right)\leq z_{n}^{j}\leq\left( 1.0-\varepsilon\right)$ is an acceptable interval.

**6. The proposed Grouping_layer**

In the $j$th layer, ${Pooling}_{j}$ specifies ${Sn}_{j}$ number of vertices as the selected vertices. The remaining vertices are the unselected vertices whose number is ${NSn}_{j}$. We named unselected vertices from the $j$th layer as a group $g_{j}$. Therefore $g_{j}$ includes $N{Sn}_{j}$ number of vertices, which aren’t selected by ${Pooling}_{j}$ and they consolidate in the $j$th group known as level in this paper. Determining the number of levels is optional and specifies the number of layers in the DGNN’s architecture. Hence, we have $J$ number of groups or levels in which the first level or group is emerged by the first layer and the last level is emerged by the last layer.

We use the g-U-Net’s architecture to extract these groups; our proposed model has a similar function to Weisfeiler-Lehman sequence of graphs ^6^. In the model proposed by Weisweiler-Lehman, a sequence of representations is generated in several steps by using a graph kernel. Similarly, we produce a sequence of representations by neural networks accompanied by their scores showing the importance of the produced representation. These representations emerged by changed graphs in several layers are generated by the proposed GCN.

In Fig. 7 $g_{1}, g_{2}, and g_{3}$ are specified for each layer in the proposed DGNN’s architecture. $g_{1}$ represents the first level; the scores or importance of $g_{1}$ is indicated by $y^{1}$ (see Supplementary Section 5) shown in Supplementary Fig. S3. $g_{2}$ represents the second level; the scores of $g_{2}$ is indicated by $y^{2}$ and $g_{3}$ represents the third level; the scores of $g_{3}$ is indicated by $z^{2}$. Therefore, the input of the *Grouping_layer* is a representation for unselected vertices and their scores that they are as follows respectively:

- $v_{j}\in\mathbb{R}^{{NSn}_{j}\times{out}_{j}},$
- $s_{j}\in\mathbb{R}^{{NSn}_{j}\times1},$

Where,$1\leq j\leq J$. Supplementary Fig. S4 shows the *Grouping_layer* architecture.

Fig. S4. An illustration of *Grouping_layer*.

We get the average of each input by using Supplementary Equation (S21) and Supplementary Equation (S22),

$vm_{j}=\frac{\left( \sum_{i\in{NSn}_{j}} v_{j}\left[ i \right] \right)}{{NSn}_{j}},$ (S21)

$sm_{j}=\frac{\left( \sum_{i\in{NSn}_{j}} s_{j}\left[ i \right] \right)}{{NSn}_{j}},$ (S22)

Where, $[\cdot]$ is a row selector operation from a matrix; $vm_{j}$ and $sm_{j}$ are mean vectors for the $j$th layer. Therefore, we have ${vm}_{j}\in\mathbb{R}^{1\times{out}_{j}}$ and $sm\in\mathbb{R}^{J\times1}$. By using Supplementary Equation (S23) and Supplementary Equation (S24), We make equal the dimensions for the whole layer of $vm$.

${v2}_{j}=\rho\left( vm_{j}\times W_{13}^{(j)}+b_{13}^{(j)} \right),$ (S23)

${v3}_{j}=\rho\left( {v2}_{j}\times W_{14}^{(j)}+b_{14}^{(j)} \right),$ (S24)

Where, $W_{13}\in\mathbb{R}^{{out}_{j}\times128}, b_{13}\in\mathbb{R}^{128}$, $W_{14}\in\mathbb{R}^{128\times64},$ and $b_{14}\in\mathbb{R}^{64}$ are trainable weight matrices. Here, activation function $\rho(\cdot)$ is tanh. In the training process, dropout is applied to ${v2}_{j}$. We have ${v3}_{j}\in\mathbb{R}^{1\times64}$. We concatenate ${v3}_{j}$ of all layers and flatten the produced matrix into a vector by using Supplementary Equation (S25) and Supplementary Equation (S26),

$v4={concatenate}_{i\in J}\left( {v3}_{i} \right),$ (S25)

$v5=FL\left( v4 \right),$ (S26)

Where, we have $v4\in\mathbb{R}^{J\times64}$. $FL\left( \cdot\right)$ is a function to flatten a matrix into a vector; here, we have $v5\in\mathbb{R}^{J*64}$. By using Supplementary Equation (S27), We define $rowscore$.

$rowscore=v5\times W_{15}+b_{15},$ (S27)

Where, $W_{15}\in\mathbb{R}^{J*64\times J} and b_{15}\in\mathbb{R}^{J}$ are trainable weight matrices. We have $rowscore\in\mathbb{R}^{J}$ including scores for all of the layers. So far, we have generated a score for each layer based on the average representation of that layer, but the flat view and using Supplementary Equation (S27) help to ensure that the score-generation process for each layer is affected by the representation generated for the other layers.

By using $sm$ defined in Supplementary Equation (S22), we define Supplementary Equation (S28) to produce the Weighted Average (WA) for each Layer.

$WA=\frac{sm}{\left( \sum_{i\in J} sm_{i} \right)},$ (S28)

Where, we have $WA\in\mathbb{R}^{J}$. Hitherto we have $J$ numbers of groups so that except for the last group, each group contains unselected words chosen by pooling layer and the last group contains the remaining words. $WA$ contains the scores assigned to each group; these scores are trained according to Supplementary Equation (S20) to place in the specific intervals from low to high (see Supplementary Section 5) so, $WA$ must be adapted to a specific pattern that is most applicable to all sentences in the database; This process helps to bring the words having representation with more valuable to level with higher score.

Now, we will calculate the relationship score based on $rowscore$ and $WA$. In the computing of the relation score, there are two texts *a* and *b* that the relationship between them must be calculated. By executing *Grouping_layer*, we obtain $rowscore$ and $WA$ for each input text, consequently:

- For input *a*, *Grouping_layer* outputs ${rowscore}_{a}$ and ${WA}_{a}$
- For input *b*, *Grouping_layer* outputs ${rowscore}_{b}$ and ${WA}_{b}$

We calculate a score for each input by defining Supplementary Equation (S29) and Supplementary Equation (S30). The score for each input is obtained by summing $rowscore$ by using the weighted average $WA$.

${score}_{a}=\sum{(WA}_{a}\circ{rowscore}_{a}),$ (S29)

${score}_{b}=\sum{(WA}_{b}\circ{rowscore}_{b}),$ (S30)

Where, ${score}_{a}\mathbb{\in R}$ and ${score}_{b}\mathbb{\in R}$. We choose the final score between ${score}_{a}\mathrm{and}{score}_{b}$ randomly (see Supplementary Equation (S31)). We propose this technique to use one of two scores to produce the ultimate result.

${score}_{final}=rand\left( {score}_{a}, {score}_{b} \right),$ (S31)

In this part, we define two losses in Supplementary Equation (S32) and Supplementary Equation (S33).

${loss}_{scorefinal}=SmoothL1Loss({score}_{a},{score}_{b}),$ (S32)

${loss}_{task}=SmoothL1Loss({score}_{final},label)$, (S33)

Due to the use of random selection in the computation of ${score}_{final}$, errors can be propagated only through ${score}_{a}$ or ${score}_{b}$. ${loss}_{scorefinal}$ allows the errors to be propagated through both ${score}_{a}\mathrm{and}{score}_{b}$; as a result, their values will be close to each other. ${loss}_{task}$ is used to close ${score}_{final}$ to the ground truth $label$.

**7. Computation of the Total Loss**

To compute the final loss, we add up all the defined losses. The total loss is deified in Supplementary Equation (S34).

${loss}_{total}=\alpha\times{loss}_{reconstraction}+KLW\times{loss}_{VAE}+{loss}_{Grouping\_layer}+{loss}_{scorefinal}+{loss}_{task}$ , (S34)

Where $\alpha$ is a coefficient for ${loss}_{reconstraction}$. We use$KLW$ indicating KLterm annealing ^7^ to avoid posterior collapse. When the decoder is too strong, VAE suffers from posterior collapse where the model learns to ignore the latent variable ^8^.$KLW$ is selected from a list generated by applying a warmup strategy in the interval [0, 1].

**8. Settings and Hyperparameters**

We apply the default configuration of RoBERTa in our experiment, except do the following list:

1. We train the transformers with learning rate 1.3e-5 and we use cosine_schedule_with_warmup ^9^ with num_warmup_steps = 200, num_cycles = 2.
2. We train the proposed layer with learning rate 1e-3, and we use cosine_schedule_with_warmup with num_warmup_steps = 50, num_cycles = 10.

Dropout is used with keep rates of 0.9. We use SmoothL1Loss and $\varepsilon=0.05$ in Supplementary Equation (S20). We set $\alpha=0.5$ in Supplementary Equation (S34). In Supplementary Section 7, we set warmup_step to 0.2.

We set the values of 𝜑 (see Supplementary Section 3) according to Supplementary Table S1.

Table S1. The values of 𝜑.

|  | *Pooling1* | *Pooling2* | *Pooling3* | *Pooling4* |
| --- | --- | --- | --- | --- |
| 2-layer system | 0.5 |  |  |  |
| 3-layer system | 0.5 | 0.5 |  |  |
| 4-layer system | 0.4 | 05 | 0.5 |  |
| 5-layer system | 0.4 | 0.4 | 0.5 | 0.5 |

We set the input and output dimensions for each layer according to Supplementary Table S2.

Table S2. The input and output dimensions of all of the layer.

|  | *input* | *output* |
| --- | --- | --- |
| layer 2 | 64 | 96 |
| layer 3 | 96 | 128 |
| layer 4 | 128 | 160 |
| layer 5 | 160 | 192 |
| layer 6 | 192 | 224 |

We feed two sentences of a sentence pair in the form of one after the other by using the specific tokens of each transformer proportional to the input form of that transformer. We set max sequence length to 128 tokens for each transformer. For creating a minibatch, we use a strategy similar to the length-based sorting strategy on the source sentence length ^10^ in the training process. We implement our proposed GCN based on the MessagePassing class provided by PyTorch Geometric ^11^.

**9. References**

1. Manning, C. D. *et al.* The Stanford CoreNLP Natural Language Processing Toolkit. in *Association for Computational Linguistics (ACL) System Demonstrations* 55–60 (2014).

2. Sennrich, R., Haddow, B. & Birch, A. Neural Machine Translation of Rare Words with Subword Units. in *Proceedings of the 54th Annual Meeting of the Association for Computational Linguistics (Volume 1: Long Papers)* 1715–1725 (Association for Computational Linguistics, 2016). doi:10.18653/v1/P16-1162.

3. Duvenaud, D. K. *et al.* Convolutional Networks on Graphs for Learning Molecular Fingerprints. in *Advances in Neural Information Processing Systems* (eds. Cortes, C., Lawrence, N., Lee, D., Sugiyama, M. & Garnett, R.) vol. 28 (Curran Associates, Inc., 2015).

4. Kipf, T. N. & Welling, M. Semi-Supervised Classification with Graph Convolutional Networks. in *ICLR 2017* (2017).

5. Girshick, R. Fast R-CNN. in *Proceedings of the IEEE International Conference on Computer Vision (ICCV)* 1440–1448 (2015).

6. Shervashidze, N., Schweitzer, P., Leeuwen, E. J. van, Mehlhorn, K. & Borgwardt, K. M. Weisfeiler-Lehman Graph Kernels. *J. Mach. Learn. Res.* **12**, 2539–2561 (2011).

7. Bowman, S. R. *et al.* Generating Sentences from a Continuous Space. in *Proceedings of The 20th SIGNLL Conference on Computational Natural Language Learning* 10–21 (Association for Computational Linguistics, 2016). doi:10.18653/v1/K16-1002.

8. Zhang, X., Yang, Y., Yuan, S., Shen, D. & Carin, L. Syntax-Infused Variational Autoencoder for Text Generation. in *Proceedings of the 57th Annual Meeting of the Association for Computational Linguistics* 2069–2078 (Association for Computational Linguistics, 2019). doi:10.18653/v1/P19-1199.

9. Transformers. *Transformers* https://huggingface.co/transformers/v2.9.1/.

10. Morishita, M. *et al.* An Empirical Study of Mini-Batch Creation Strategies for Neural Machine Translation. in *Proceedings of the First Workshop on Neural Machine Translation* 61–68 (Association for Computational Linguistics, 2017). doi:10.18653/v1/W17-3208.

11. PyTorch Geometric. *GitHub* https://github.com/rusty1s/pytorch_geometric.
